# Mitomycin C treatment improves pancreatic islet graft longevity in intraportal islet transplantation by suppressing proinflammatory response

**DOI:** 10.1038/s41598-020-69009-8

**Published:** 2020-07-21

**Authors:** Kei Yamane, Takayuki Anazawa, Seiichiro Tada, Nanae Fujimoto, Kenta Inoguchi, Norio Emoto, Kazuyuki Nagai, Toshihiko Masui, Hideaki Okajima, Kyoichi Takaori, Shoichiro Sumi, Shinji Uemoto

**Affiliations:** 1 Department of Surgery, Graduate School of Medicine, Kyoto University, Kyoto, 6068507 Japan; 2 Department of Paediatric Surgery, Kanazawa Medical University, Kanazawa, 9200293 Japan; 3 Laboratory of Organ and Tissue Reconstruction, Institute for Frontier Medical Sciences, Kyoto University, Kyoto, 6068507 Japan

**Keywords:** Chemokines, Allotransplantation

## Abstract

The in vitro culture period prior to cell transplantation (i.e. pancreatic islet transplantation) enables cell modification and is thus advantageous. However, the islet preconditioning method has not been fully explored. Here we present a simple approach for islet preconditioning that uses the antibiotic mitomycin C (MMC), which has antitumor activity, to reduce islet immunogenicity and prevent proinflammatory events in an intraportal islet transplantation model. Freshly isolated mice islets were treated for 30 min with 10 μg/mL MMC or not, cultured for 20 h and transplanted into the livers of syngeneic or allogeneic diabetic mouse recipients. In the allogeneic model, MMC preconditioning significantly prolonged graft survival without requiring immunosuppressants. In vitro, MMC treatment suppressed the expression of proinflammatory cytokines in islet allografts, while immunohistochemical studies revealed the suppression of inflammatory cell infiltration into MMC-treated allografts relative to untreated allografts. Furthermore, MMC preconditioning significantly suppressed the mRNA expression of proinflammatory cytokines into the transplant site and induced the differentiation of regulatory T cells with the ability to suppress CD4^+^ T cell-mediated immune responses. In conclusion, islet preconditioning with MMC prolonged graft survival in an intraportal islet transplantation model by suppressing proinflammatory events and inducing potentially regulatory lymphocytes.

## Introduction

Pancreatic islet transplantation is considered a safe and effective treatment that significantly improves the health-related quality of life of patients with an impaired awareness of hypoglycaemia who experience severe hypoglycaemic events^1,2^. Still, despite significant progress in the field of clinical islet transplantation, multiple donors are needed to achieve insulin independence^3,4^. Moreover, the rate of insulin independence declines markedly each year after islet infusion^5,6^, primarily because islet engraftment and survival are strongly inhibited by factors such as instant blood-mediated inflammatory reactions (IBMIR)^7^, early innate immune responses^8^ and allogeneic rejection^5,9^.

Recent improvements in islet transplantation rate have been attributed to refinements in immunosuppressive therapies^6^. However, immunosuppressive drugs are associated with several adverse side effects, including myelosuppression, infection and malignancy^10^. Moreover, these drugs are directly toxic to islets^5,11,12^. Therefore, a strategy for protecting islet allografts against immune responses in the absence of immunosuppressive drugs would constitute an ideal approach to islet transplantation.

Several centres subject human islets to a culture period before transplantation^13^. This period provides an opportunity for cell modifications intended to reduce immunogenicity, a process known as preconditioning. The greatest advantage of preconditioning is the ability to reduce the dose of the immunosuppressive agent. Hence, optimal preconditioning would help the recipients of islet allografts to avoid the adverse effects of immunosuppressive drugs. To date, various preconditioning approaches have been reported to improve islet engraftment. For example, Lau et al. reported a prolonged engraftment time for rat allografts preconditioned with ultraviolet irradiation^14^, while Gotoh et al. showed that gamma-irradiation could prolong the survival of islet allografts and xenografts in recipient mice^15^. Other reports demonstrated that the pre-treatment and subsequent culture of donor islets with mitomycin C (MMC), an antibiotic with antitumor activity, significantly prolonged graft survival in a mouse model of renal subcapsular islet transplantation^16,17^.

The above-described preconditioning methods have been applied only in mouse models of renal subcapsular islet transplantation. However, this transplant site is not suitable for preclinical studies of islet infusion because of differences in the innate and alloimmune systems; rather, the liver is considered more appropriate^18^. In clinical settings, islet allografts are transplanted into the liver via the portal vein. Therefore, preclinical islet transplantation data should be obtained from mice following intraportal islet transplantation. Furthermore, previous studies have not fully elucidated the mechanism by which MMC induces prolonged engraftment. The aims of this study are to test our hypothesis that the efficacy of MMC preconditioning, which prolongs islet allograft survival in renal subcapsular islet transplantation, can be applied to intraportal islet transplantation as well as to elucidate its mechanism.

## Results

### MMC preconditioning prolongs islet allograft survival after intraportal islet transplantation

**Figure 1:**
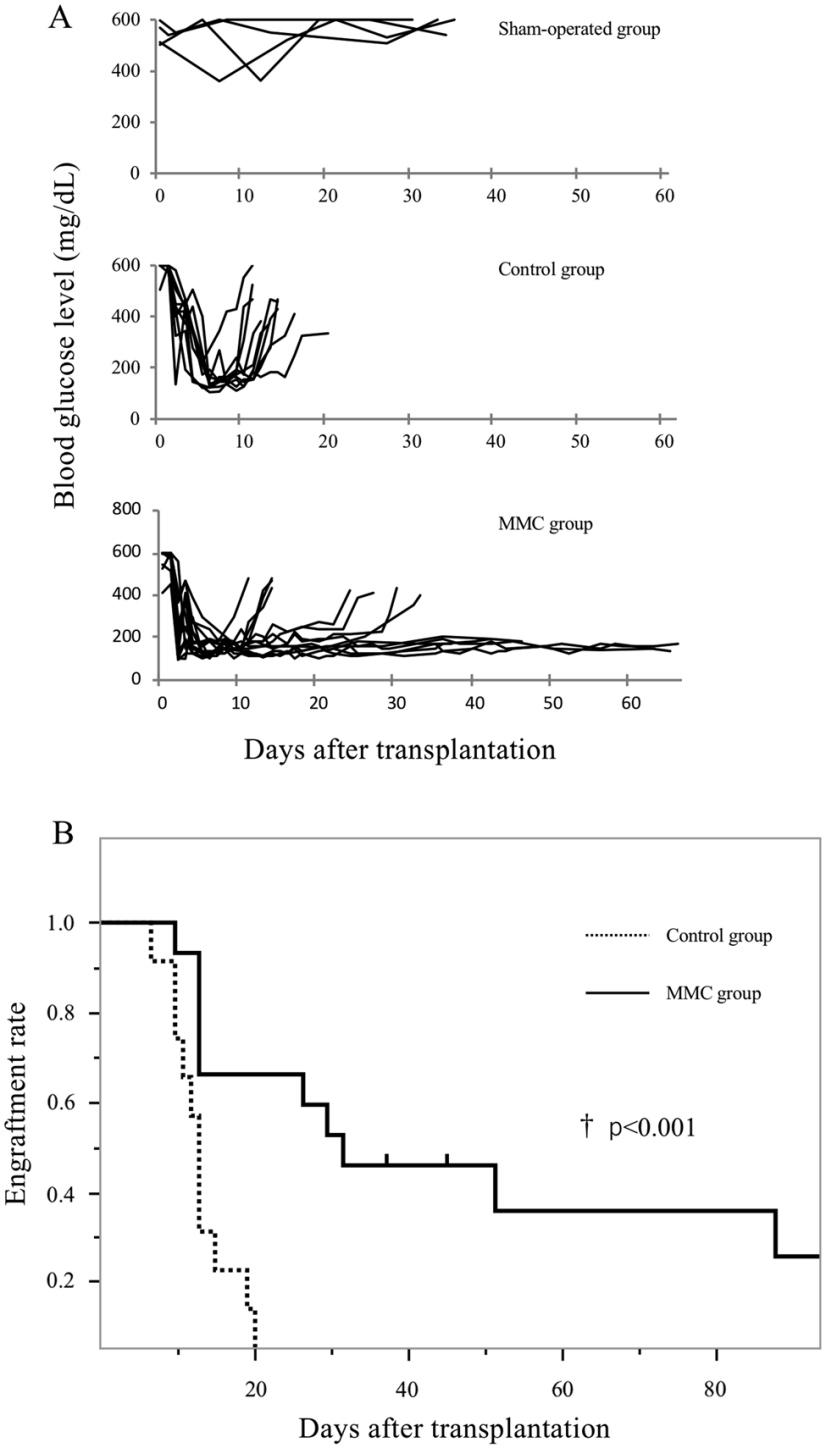
Effect of mitomycin C (MMC) preconditioning on islet allograft engraftment. (**A**) Blood glucose levels of mice that received saline only (sham-operated group, n = 4), nontreated islets (control group, n = 11), or MMC preconditioning islets (MMC group, n = 14). (**B**) Kaplan–Meier survival analysis of the control and MMC groups. MMC preconditioning significantly prolonged the islet survival time (p < 0.001).

We initially evaluated the graft survival times in the control and MMC groups to assess the effects of MMC preconditioning before intraportal islet allotransplantation. All the recipients in both groups achieved normoglycaemia within 5 days after transplantation with 600 islets, whereas recipients in the sham-operated group maintained a high glucose level > 30 days after the operation. However, all recipient mice in the control group acutely rejected the islet allografts, whereas MMC preconditioning prolonged islet allograft survival (Fig. 1A,B; p < 0.001). The median islet survival durations in the control and MMC groups were 13 and 28 days, respectively. Notably, 7 of 14 mice (50%) in the MMC group maintained normoglycaemia > 30 days without any immunosuppressive drug administration. An immunofluorescence study of the pancreases of normal mice and transplant recipient mice was performed to ensure that euglycemia was due to insulin secreted by the islet grafts (Supplementary Fig. S1). The percentage of the total area that was anti-insulin positive was significantly lower in the pancreatic tissue isolated from STZ treated mice than in tissue from normal mice; likewise, the islet number per unit area of pancreatic tissue was significantly lower in mice treated with STZ than in the normal mice (Supplementary Fig. S2).

### Efficacy of MMC preconditioning in a syngeneic model

To evaluate the ability of MMC preconditioning to suppress early graft loss, a secondary examination was performed to determine whether preconditioning improved the engraftment rate in an intraportal syngeneic model. However, a comparison of the engraftments between the control and the MMC groups revealed no significant difference in the number of transplanted islet cells (Table 1). Moreover, the days to normoglycemia did not differ significantly between the control and the MMC groups. Therefore, this syngeneic model did not confirm the efficacy of MMC preconditioning.

**Table 1:** Results of islet transplantation in a syngeneic model.

		No. of animals	Engraftment	Engraftment rate (%)	Days to engraftment (days)
250 islets	Control	3	2	66.7	7, 5
	MMC (+)	4	3	75.0	13, 9, 6
200 islets	Control	6	4	66.7	10, 13, 7, 8
	MMC (+)	5	3	60.0	13, 13, 6, 11
175 islets	Control	6	3	50.0	8, 12, 10
	MMC (+)	7	3	42.9	7, 9, 6
150 islets	Control	8	1	12.5	21
	MMC (+)	4	1	25.0	25

A comparison of engraftment rates between the control and mitomycin C (MMC) groups revealed no significant difference in the number of transplanted islet cells.

### Assessment of the in vivo function of islet allografts

**Figure 2:**
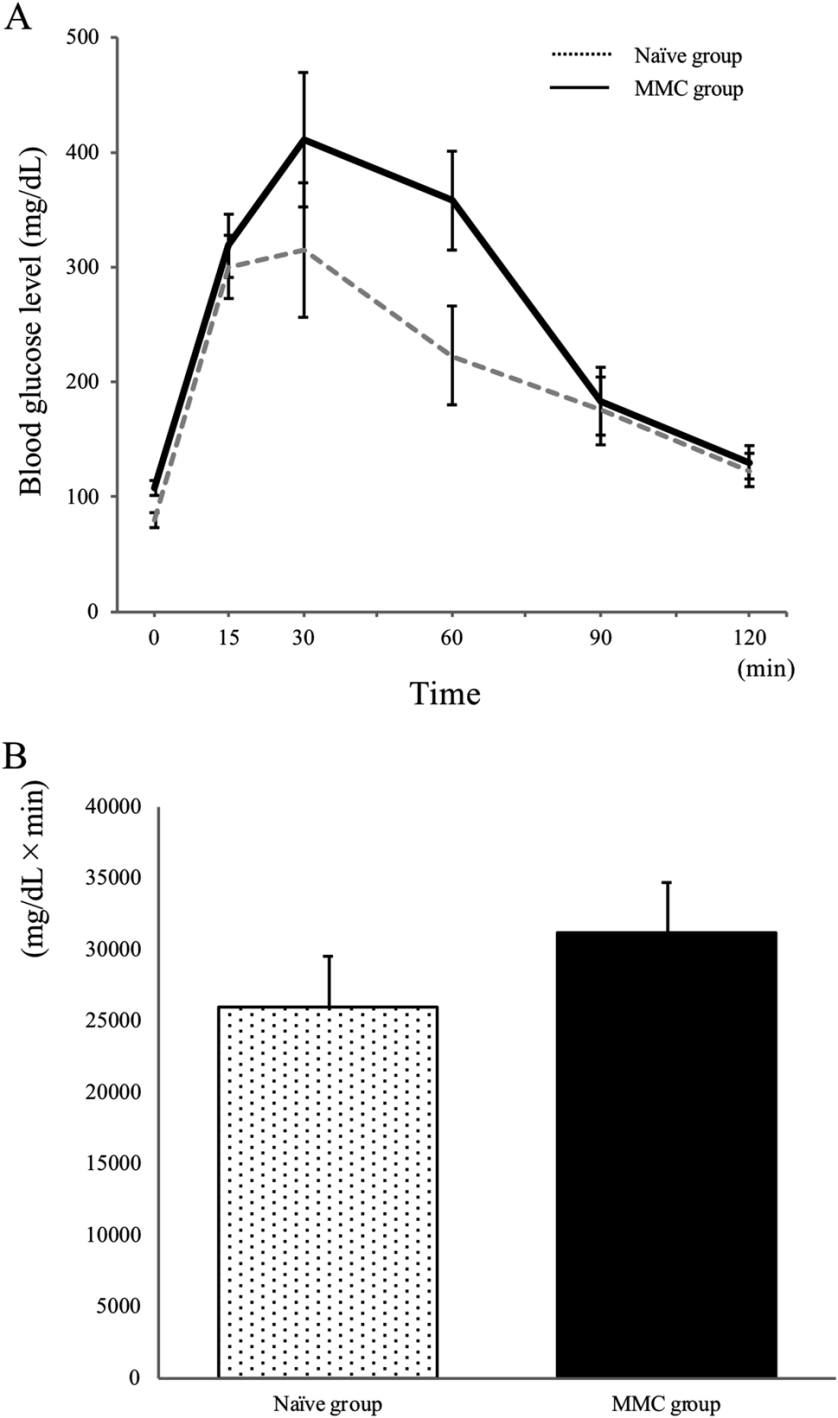
Intraperitoneal glucose tolerance test results for normal (naïve group, n = 3) and mitomycin C treated mice (MMC group, n = 3) (**A**). The area under the curve was calculated to compare the glycaemic responses between groups (**B**). No significant difference was identified between the groups.

We subjected normal healthy mice (naïve mice group) and MMC mice (MMC group) that maintained normoglyacemia 30 days after transplantation to an intraperitoneal glucose tolerance test (IPGTT; Fig. 2A,B; n = 3 per group). A blood glucose curve indicated good islet function in vivo in the MMC group. Neither the blood glucose curves nor the areas under the curves differed significantly between the MMC and the naïve mice groups.

### MMC preconditioning changes the inflammatory gene expression of islet allografts

**Figure 3:**
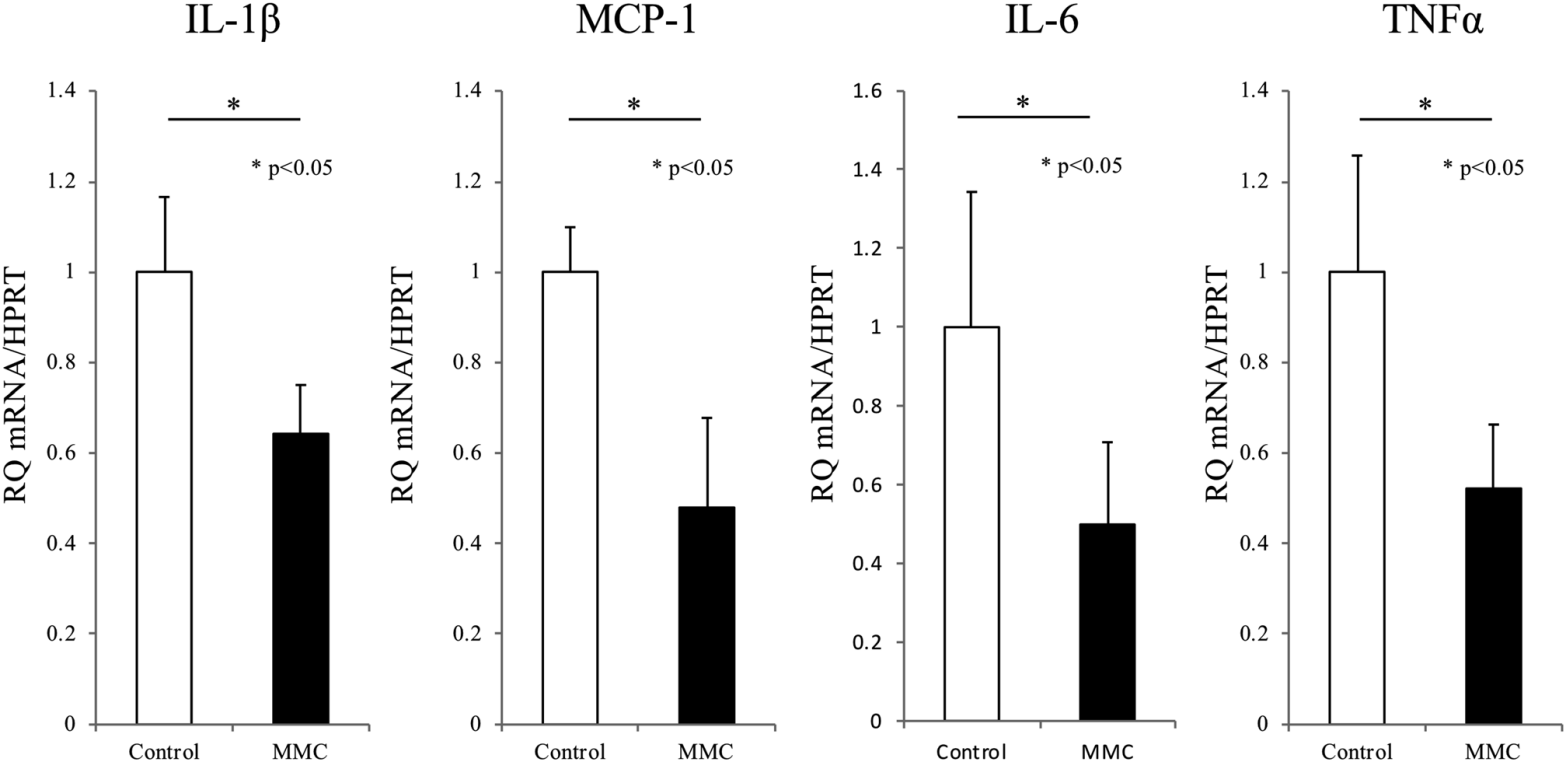
Mitomycin C (MMC) preconditioning suppressed inflammatory gene expression in the allografted islets. The islets were subjected to mRNA extraction after a 24-h culture. Notably, MMM preconditioning suppressed the expression of interleukin (IL)-1beta, monocyte chemoattractant protein (MCP)-1, IL-6 and tumour necrosis factor (TNF)-α mRNA (p < 0.05, n = 4 for all). Hypoxanthine phosphoribosyltransferase (HPRT) mRNA was used as an internal control.

Next, we evaluated whether MMC preconditioning altered the expression of inflammatory genes in the islet allografts. A quantitative real-time polymerase chain reaction (qRT-PCR) analysis revealed significantly reduced expression of the genes encoding interleukin (IL)-1β, monocyte chemoattractant protein (MCP)-1, IL-6 and tumour necrosis factor (TNF)-α in MMC-preconditioned islets, compared with control islets (p < 0.05 for all; Fig. 3).

### MMC preconditioning suppresses cellular infiltration to allografts and inflammation at the transplant site

**Figure 4:**
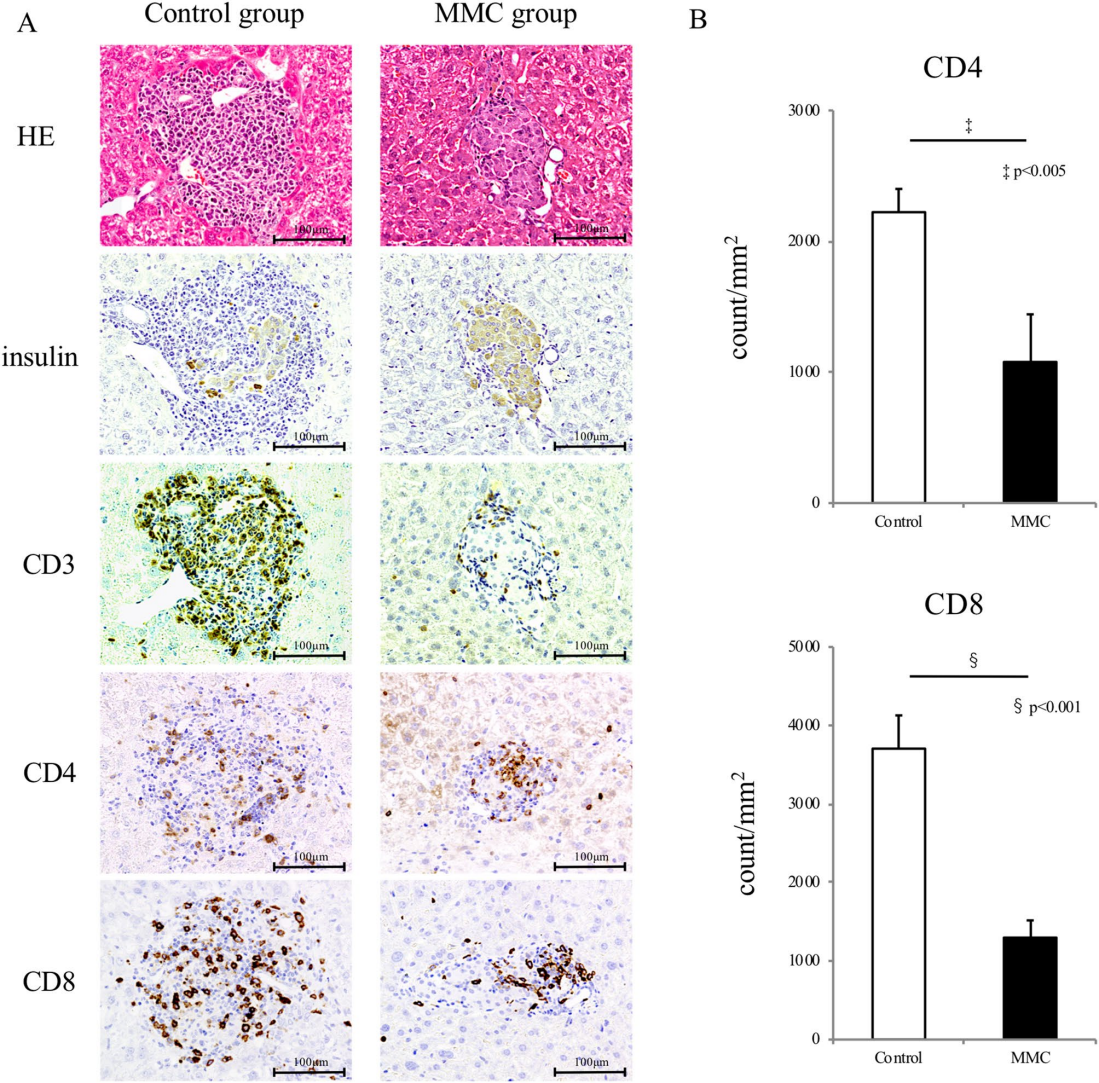
Immunohistological study of islet allografts harvested from mice in the control and mitomycin C (MMC) groups at 7 days after transplantation. Islet allografts in the recipient livers were stained with haematoxylin–eosin and antibodies specific for insulin, CD3, CD4 and CD8 (**A**). CD4^+^ and CD8^+^ cells surrounding and infiltrating the allograft were counted (two sections per animal, 4 animals) and those numbers per islet area were compared between the control and MMC groups (p < 0.005 and p < 0.001, respectively, n = 8) (**B**).

At 7 days after transplantation, the transplanted islet allografts in both groups were assessed immunohistologically. Severe cellular infiltration, into and surrounding the islet allografts were noted in the control group tissues. The majority of infiltrating cells were CD3^+^ T cells. Moreover, allograft failure was detected, and the insulin-positive area had decreased. Conversely, the MMC-treated allografts contained intact insulin-stained cells with minimal inflammatory cell infiltration (Fig. 4A). The CD4^+^ and CD8^+^ cell counts in MMC-treated allografts were significantly higher than those in the nontreated allografts.

**Figure 5:**
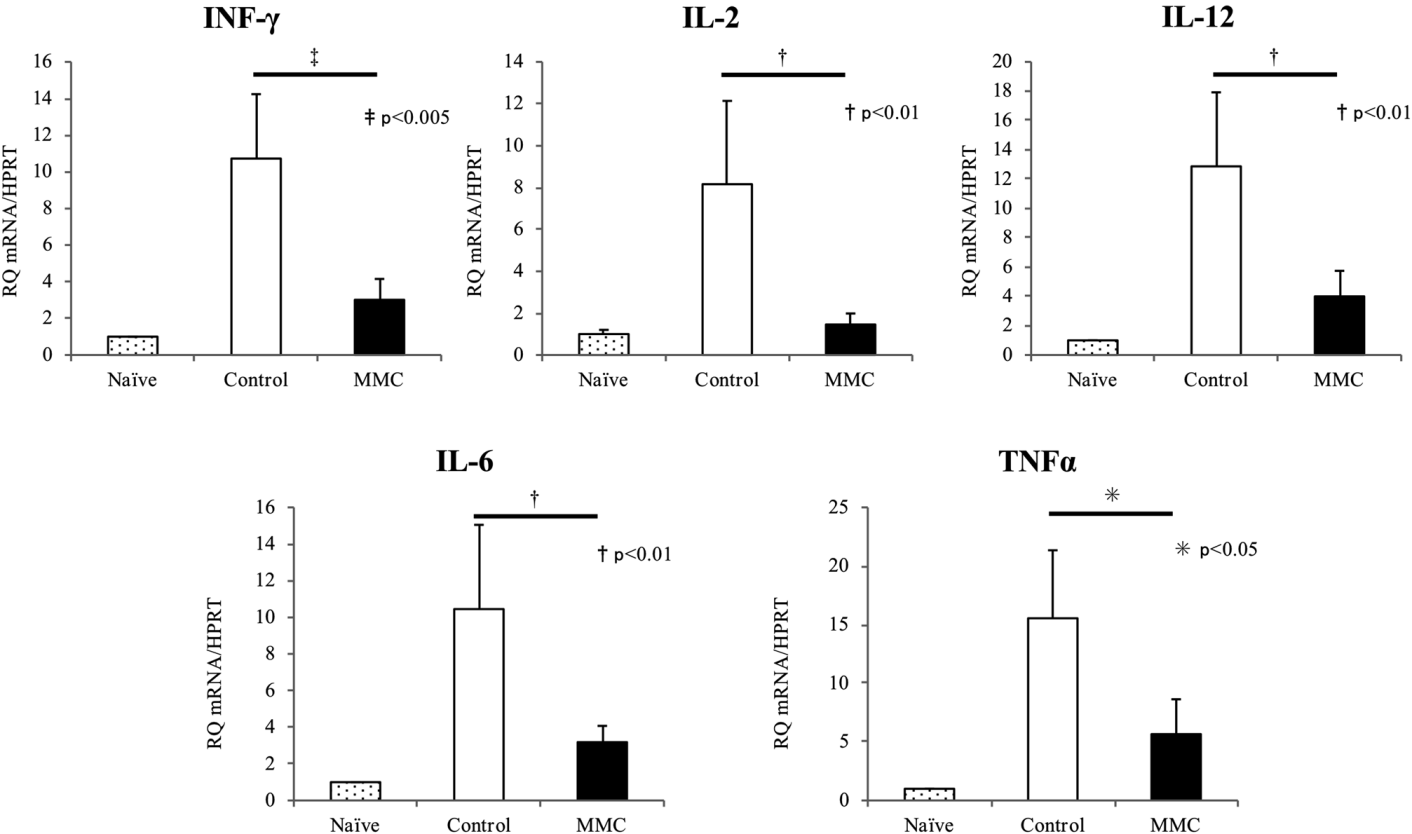
Mitomycin C (MMC) preconditioning inhibited intrahepatic inflammatory responses. (**A**) Liver samples were extracted from recipients 7 days after transplantation and from healthy mice. The expression of interferon (INF)-γ, interleukin (IL)-2, IL-12, IL-6 and tumour necrosis factor (TNF)-α mRNA in the recipient livers was measured using quantitative RT-PCR (n = 4). (**B**) INF-γ levels in the transplanted livers (extracted 7 days after transplantation) of the control and MMC groups were measured by ELISA. INF-γ levels were significantly lower in the livers of the MMC group than in those of the control group (n = 4; p < 0.01).

After intraportal transplantation, the in vivo inflammatory responses at the transplant site were assessed using qRT-PCR. Liver samples were obtained from recipient animals in both groups at 7 days after transplantation and compared with liver samples from naïve mice. Notably, the expression of interferon-γ, IL-6, TNF-α, IL-2 and IL-12 mRNA was increased in the liver tissues from both the control and MMC groups, compared with the naïve liver tissues (Fig. 5A). However, expression of the indicated genes was significantly suppressed in livers from the MMC group, compared with those from the control group. Additionally, we measured INF-γ levels in the transplanted livers of the control and MMC groups by ELISA. INF-γ levels were significantly lower in the livers of the MMC group than in those of the control group (Fig. 5B, n = 4; p < 0.01).

### Analysis of CD3^+^ T cells at the transplant site

**Figure 6:**
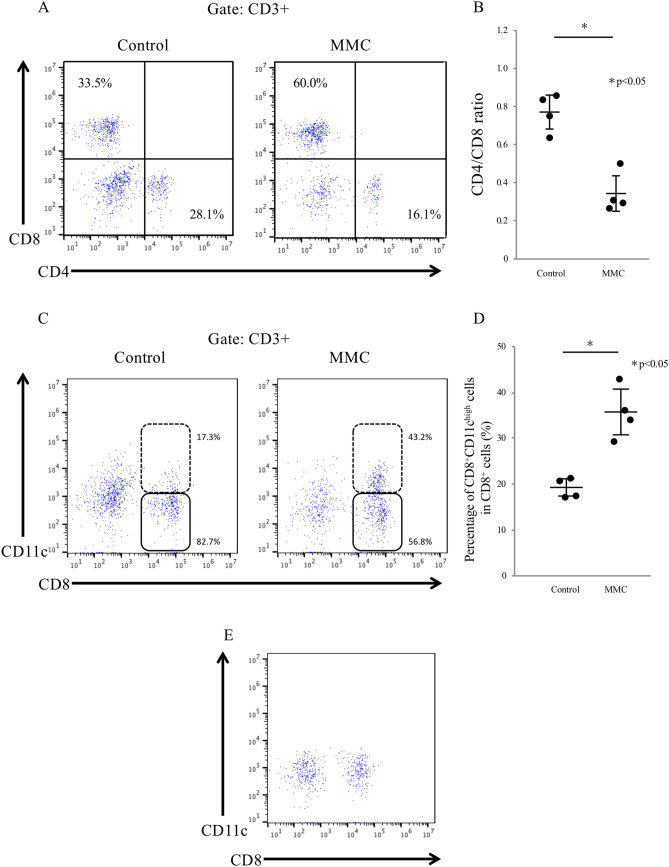
Intrahepatic lymphocytes were isolated from transplant recipients in the control and mitomycin C (MMC) groups 7 days after transplantation and subjected to flow cytometric analysis. The numbers of CD4^+^, CD8^+^ and CD11c^+^ cells were compared between the control and MMC groups (**A**–**D**, n = 4). Naïve mouse liver tissue was used as a control (E).

Next, hepatic nonparenchymal cells, including hepatic lymphoid cells, were isolated 7 days after transplantation and assessed using flow cytometric analysis to evaluate whether MMC preconditioning affected the types of immune cells that had infiltrated the transplant site. Specifically, we focused on CD3^+^ T cells and the CD4^+^/CD8^+^ cell ratio at the transplant site. Compared with the control livers, the MMC group livers exhibited a significant expansion of the CD8^+^ T cell population, which then dominated the CD4^+^ T cells, with respective CD4/CD8 ratios of 0.77 ± 0.05 and 0.34 ± 0.05, respectively (p < 0.001; Fig. 6A,B). A further analysis of the CD8^+^ T cells indicated a significant increase in the number of CD8^+^CD11c^high^ cells, which are known to suppress CD4 + T cell activity and promote immunosuppression in vivo^19^, in MMC group livers (Fig. 6C). The percentages of CD8^+^CD11c^high^ cells among the total CD8^+^ cells were 35.7% ± 2.8% in the MMC group livers and 19.3% ± 1.1% in the control group (p < 0.05; Fig. 6D). Conversely, few CD8^+^CD11c^high^ cells were detected in the naïve mice livers (Fig. 6E). Moreover, few traditional T regulatory cells (CD4^+^CD25^+^Foxp3^+^ T cells) were observed around the livers of mice in the control and MMC groups (data not shown).

## Discussion

Our study results demonstrated that MMC preconditioning prolongs the survival of islet allografts in a mouse model of intraportal islet transplantation. Surprisingly, half of the recipients that received MMC-preconditioned islet allografts maintained normoglycemia > 30 days without additional treatment. Moreover, the in vitro study suggested that MMC preconditioning suppressed the release of proinflammatory cytokines from islet allografts and the infiltration of host immune cells into the allografts. In addition, the population of immunosuppressive CD8^+^CD11c^high^ cells was increased following intraportal transplantation with MMC-preconditioned islets. Notably, ours is the first report to establish the efficacy and mechanism of MMC preconditioning in the context of intraportal islet transplantation.

Previous reports showed that MMC-preconditioned islets had prolonged survival durations in both a mouse-to-mouse allotransplant model^16^ and a rat-to-mouse xenotransplant model^17^. Those studies did not administer immunosuppressive agents to the recipients, consistent with our present study. However, those studies used a renal subcapsular transplant model. A change in the transplant site would expose the islet allografts to different immune responses. Our study is thus notable, as it is the first to demonstrate the efficacy of MMC preconditioning in an intraportal transplant model. Previously, Mellgren et al. used a ^3^H-thymidine incorporation assay to evaluate and compare islet grafts transplanted into the renal subcapsular, intraportal and intrasplenic spaces and demonstrated that the former site best supported the proliferation of grafted islet cells^20^. In contrast, Kim et al. reported that the subcapsular site was most effective for islet transplantation because it was associated with the lowest marginal mass and shortest mean time to reach euglycemia, compared with other sites in the liver, muscle and omentum^21^. Further, Lacy et al. demonstrated more rapid rejection of rat, hamster and rabbit islet xenografts transplanted into the mouse liver via the portal vein, compared with those transplanted into the kidney capsule^22^. Moreover, the site-dependent activation of innate immune cells may initiate and amplify allospecific adaptive immune responses^23^, which may account for the differences in cellular immune responses between the kidney capsule and the liver. As human islet allografts are transplanted into the liver via the portal vein in clinical settings, preclinical studies are needed to confirm the real-life efficacy of intraportal islet transplantation.

Previous studies have not fully elucidated the mechanism by which MMC prolongs engraftment. At the beginning of this experiment, we presumed that MMC preconditioning was effective both for syngeneic and allogeneic islet transplantation. Accordingly, we attempted to demonstrate the effect of MMC precondition on allorejection in vivo using syngeneic and allogeneic models. In the latter model, we could establish the prolonged engraftment of MMC-preconditioned islet allografts. However, MMC preconditioning did not improve the engraftment rate in the syngeneic model (Table 1), suggesting that MMC preconditioning does not have a significant effect on non-alloimmune responses (e.g. IBMIR). Therefore, the prolongation of islet allograft was not induced by suppressed non-specific responses but was induced by other mechanisms.

Inflammatory gene expression is upregulated during the islet isolation and culture period, with negative effects on the engraftment of islet allografts^24^. Sato et al. reported contrasting changes in gene expression between MMC-preconditioned islets and those subjected to culture only. Specifically, the expression of genes involved in various aspects of cell activation, such as cellular movement, immune cell trafficking and inflammatory responses, was downregulated in MMC-conditioned islets^25^. Moreover, we demonstrated that MMC preconditioning significantly suppressed the expression of IL-1β, MCP-1, IL-6 and TNF-α in islet allografts. Previous reports demonstrated a unique immunomodulatory approach to targeting proinflammatory cytokines such as IL-1β^26^, TNF-α^27^, MCP-1^28^ and IL-6^29,30^ and their respective receptors, and reported that the blockade of even one proinflammatory cytokine could prolong the survival of the transplanted islets. Our data, however, indicated that MMC preconditioning could induce the suppression of multiple cytokines, which was associated with poor outcomes^24^. This is one of the mechanisms by which MMC preconditioning promotes islet allograft longevity.

T cells play a key role in allograft rejection. Accordingly, several T cell depletion strategies have been attempted to prolong graft survival^31,32^. IL-12, which is primarily produced by activated macrophages, induces the differentiation of CD4^+^ Th1 cells^33,34^ that secrete Th1-mediated cytokines, such as IL-2 and INF-γ, to promote the proliferation of activated T cells. In other words, these cytokines induce hypersensitivity and inflammation and trigger CD8^+^ T cells to attack the allografts directly^35^. In our study, several populations of T cells appeared to be suppressed in the MMC-treated livers, which expressed significantly lower levels of IL-2, INF-γ, IL-12, TNF-α and IL-6 mRNAs, compared with the control livers. Furthermore, our histologic analysis indicated reduced CD3^+^ T cell aggregation around the MMC-preconditioned islet allografts, compared with the control allografts. Taken together, these results suggest a prolonged engraftment time in the MMC group.

We additionally observed a difference in the ratio of CD4^+^/CD8^+^ cells at the transplant site between the control and MMC-preconditioned groups, and an increased number of CD8^+^CD11c^high^ cells among the total CD8^+^ cell population in liver tissues from the latter group. CD8^+^CD11c^high^ T cells suppress inflammation by killing activated CD4^+^ T cells^19,36^, consistent with our observation of a decreased CD4^+^ cell population and increased CD8^+^CD11c^high^ cell population at the transplant site in the MMC group. These findings suggest that MMC-preconditioned islets induce the differentiation of CD8^+^CD11c^high^ cells and thus suppress allorejection.

MMC is an antibiotic agent with genotoxic effects both in vitro and in vivo^37^. Saito et al. reported that MMC preconditioning significantly increased the rate of islet recovery after culture and significantly reduced central damage to the islets during culture^38^. Moreover, Saito and colleagues demonstrated the importance of p53 and p21^waf1^ activation, consistent with several studies that reported the ability of MMC to induce apoptosis^39–41^. However, no previous studies investigated whether MMC preconditioning affected the role of the islet as an insulin-secreting cell. In this study, our in vivo IPGTT data indicated that the MMC-preconditioned islet allografts secreted sufficient amounts of insulin to reduce the blood glucose levels in diabetic mice. In other words, MMC preconditioning preserved the islet cells and did not diminish the role of the islet as an insulin-secreting cell.

MMC preconditioning has been used previously to improve the outcomes of various organ and cell transplantation procedures. For example, Goto et al. reported a prolonged hepatic allograft engraftment time following the injection of MMC-treated donor splenocytes 7 days before transplantation in the rat model^42^. Liu et al. reported a prolonged survival time for MMC-preconditioned heart allografts^43^, and demonstrated that the co-injection of MMC-preconditioned splenocytes and an MMC-preconditioned heart allograft led to a prolonged engraftment time, compared with the injection of a preconditioned allograft alone. In those reports, MMC-preconditioned donor cells (e.g. splenocytes) were thought to induce donor-specific immune regulation. In our study, the islet allografts were preconditioned with MMC in the presence of contaminants, such as lymphocytes, because, in clinical setting, isolated islets and contaminants are co-transplanted into the recipient’s liver. Consequently, these preconditioned contaminants were co-transplanted with the MMC-preconditioned islet allografts. Possibly, these MMC-preconditioned contaminants, especially immune cells, could induce donor-specific tolerance.

As described above, various mechanisms prolong the engraftment of MMC-preconditioned islet allografts. Here, we demonstrated that MMC preconditioning could suppress the mRNA expression of proinflammatory cytokines from islet allografts, thus protecting them from immune attacks. Moreover, the mRNA expression of cytokines promoting the proliferation of activated T cells was suppressed at the sites where MMC-preconditioned islets were transplanted. Together with the lymphocyte-mediated induction of a negative inflammatory response, this response prolonged the engraftment of islet allografts without requiring immunosuppressive drugs.

The present study had several limitations. First, although rodent and human islets are similarly sized, the liver structure and vasculature exhibit vast interspecies anatomical differences. Therefore, the present results obtained with mouse islets are not entirely translatable to islet transplantation in a clinical setting. Previous studies are needed to determine whether MMC preconditioning would be similarly effective for the transplantation of islets from other animals and humans. Still, our confirmation of the ability of MMC to inhibit the alloimmune response in an animal lends support to the clinical use of this preconditioning protocol. Second, the mechanism by which MMC-preconditioned islet allografts induces the differentiation of regulatory CD8^+^CD11c^high^ T cells has not been fully elucidated. We speculate either that some cytokines secreted from MMC-treated islets induce CD8^+^CD11c^high^ cells or that MMC preconditioning directly induces CD8^+^CD11c^high^ T cells from the immunological cells in islets or contaminants. In some reports, these T cells were reported to act as effectors in immune homeostasis^44^. A further investigation is needed to evaluate the induction of regulatory cells by MMC preconditioning. Finally, other as-yet undetermined mechanisms may prolong graft survival. Additional studies of MMC preconditioning would, therefore, further the clinical application of this procedure in other animals and humans.

In conclusion, MMC preconditioning (i) can prolong the survival of an intraportal islet transplant by inhibiting proinflammatory events and (ii) can promote the infiltration of host immune cells responsible for suppressing immune responses to islet grafts. Our findings further suggest that MMC preconditioning might induce the differentiation of potentially regulatory lymphocytes. Our findings may contribute to the establishment of a strategy for successful clinical islet transplantation with a reduced dose of immunosuppressive agents.

## Methods

### Experimental protocols

We evaluated the efficacy of MMC preconditioning in two models: an intraportal and syngeneic islet transplantation model, and an allogeneic model. In the former model, B6 mice were used as both donors and recipients. In the latter model, BALB/c mice were used as donors, and B6 mice were used as recipients. In both models, recipients were divided into two groups: the MMC group, which received MMC-preconditioned islets, and the control group, which received nontreated islets. A third sham-operated group, in which mice received only saline via the portal vein, was used to confirm whether the transplanted islets reduced the recipients’ blood sugar levels. The graft survival duration and engraftment rate were compared between the two experimental groups. Moreover, recipient livers were harvested from allogeneic model mice at 7 days after transplantation and were subjected to a histologic analysis, qRT-PCR and a flow cytometric analysis specific for infiltrating lymphoid cells.

### Pancreatic islet isolation

The pancreatic islet isolation method was conducted as previously described^45^. Briefly, donor BALB/c and B6 mice were anaesthetised by isoflurane inhalation, and the common bile duct was clamped at the entrance to the duodenum. Hanks’ balanced salt solution supplemented with 0.15 mg/mL collagenase P (Roche Diagnostics, Indianapolis, IN) was injected into the common bile duct to distend the pancreas. Subsequently, the pancreas was excised, incubated at 37 °C for 18 min, washed twice and purified via discontinuous density gradient centrifugation (1.110, 1.103, 1.096 and 1.070 g/mL). ET-Kyoto solution (Otsuka Pharmaceutical, Tokyo, Japan) and OptiPrep solution (Axis-Shield, Oslo, Norway) were used to produce the gradients. Each distinct layer of islets was collected and washed after centrifugation.

### Induction of diabetes mellitus in the recipient mice

Seven days before transplantation, a single intraperitoneal injection of streptozotocin at a dose of 150 mg/kg body weight was administered to B6 mice to induce diabetes mellitus. Subsequently, blood samples were collected via the tail vein, and blood glucose levels were measured using an Accu-Chek blood glucose monitor (Roche Diagnostics K.K., Tokyo, Japan). Mice with two consecutive measured blood glucose concentrations of 450 mg/dL were used as recipients.

### MMC preconditioning and islet transplantation

The islets were isolated, hand-selected and divided into two groups. MMC (FUJIFILM Wako Pure Chemical Corporation, Osaka, Japan) preconditioned islets were incubated in RPMI supplemented with 10 μg/mL MMC for 30 min^16,17^, while nontreated islets were incubated in RPMI alone for the same amount of time. Subsequently, the islets were washed twice and cultured in RPMI supplemented with 100 U/mL penicillin, 100 U/mL streptomycin and 10% foetal bovine serum for 20 h at 37 °C in a humidified atmosphere of 5% CO_2_ and 95% air. Next, the islets were transplanted into the recipient livers via the portal vein. Recipients in the allogenic model received 600 islets, while recipients in the syngeneic model received 150, 175, 200 or 250 islet isografts. No recipients received immunosuppressive agents. The blood glucose concentrations of all recipient mice were monitored. Normoglycaemia was defined as a blood glucose level of < 200 mg/dL for three consecutive days, while specimen rejection was defined as a blood glucose level > 350 mg/dL.

### Intraperitoneal glucose tolerance test

To investigate the functions of transplanted islets, MMC mice in the allogeneic model were subjected to an IPGTT at 30 days after islet transplantation. The mice were fasted for 16 h before the start of the examination. Thereafter, recipient mice received an intraperitoneal injection of normal saline containing 2.0 g/kg body weight of glucose, and the blood glucose levels were measured before and at 15, 30, 60, 90 and 120 min after the injection. Using the same method, naive B6 mice were evaluated as controls.

### qRT-PCR analysis

Total RNA was extracted from the cultured islets immediately after the 20-h culture and from liver samples, using the PureLink RNA Mini Kit (Invitrogen, Tokyo, Japan). Total RNA was reverse transcribed to complementary DNA using the SuperScript VILO cDNA Synthesis Kit (Invitrogen). Next, we performed qRT-PCR using gene-specific PCR primers (Supplementary Table S1), PowerUp SYBR Green Master Mix and the StepOne Real-Time PCR System (Applied Biosystems, Foster City, CA, USA). Each measurement was performed in triplicate. All gene expression levels were normalised to the internal control (hypoxanthine phosphoribosyltransferase). Fold changes in the gene expression levels were calculated using the 2^−ΔΔCT^ method^46^.

### Measurement of INF-γ in the transplant site

Liver samples from transplanted recipients excised 7 days after transplantation were manually homogenised in Tissue Protein Extraction Reagent (Thermo Fisher Scientific, Waltham, MA, USA) supplemented with protease inhibiter (Halt Protease Inhibitor Cocktail; Thermo Scientific). INF-γ was measured in duplicate using a mouse INF-γ ELISA kit (BD Biosciences, San Jose, CA, USA).

### Histologic study

In both groups, the excised livers containing islet grafts were fixed in 10% formaldehyde, embedded in paraffin and stained with haematoxylin–eosin. Immunohistochemical staining was performed as previously reported^47^. The sections were incubated overnight at 4 ℃ with the following primary antibodies: mouse anti-insulin (1:2000 dilution, 66198-1-Ig; Proteintech Japan, Tokyo, Japan), rabbit anti-CD3 (1:100 dilution, Ab16669; Abcam, Cambridge, UK), rabbit anti-CD4 (1:100 dilution, 25229; Cell Signaling Technology, Inc., Danvers, MA, USA) and rabbit anti-CD8 (1:400 dilution, 98941; Cell Signaling Technology). Subsequently, the sections were incubated with a biotinylated second antibody diluted to 1:300 in phosphate-buffered saline (PBS) for 40 min, followed by washes in PBS (six times, 5 min). Next, an avidin–biotin-peroxidase complex (ABC-Elite, Vector Laboratories, Burlingame, CA, USA) at a dilution of 1:100 in bovine serum albumin was applied for 50 min. After washing in PBS, 3,3′-diaminobenzidine was used to induce the colorimetric reaction. The nuclei were counterstained with haematoxylin. CD4^+^ and CD8^+^ cells were counted using ImageJ software (US National Institutes of Health, Bethesda, MD, USA).

### Isolation of hepatic lymphoid cells and flow cytometric analysis

Recipient livers were excised, minced into small fragments and passed through steel mesh with a gauge of 200 μm. The resulting suspension was washed with PBS, and the hepatic lymphoid cells were isolated via discontinuous density gradient centrifugation with Ficoll-Paque PREMIUM solution (GE Healthcare, Little Chalfont, UK). The hepatic lymphoid cells were stained with FITC-conjugated anti-CD3 (17A2; Biolegend, San Diego, CA, USA), PE-conjugated anti-CD4 (RM4-4; Biolegend), APC-conjugated anti-CD8a (53-6.7; Biolegend) and PE-conjugated anti-CD11c (N418; Biolegend). Three-color flow cytometric analysis was conducted using a BD Accuri C6 flow cytometer (BD Biosciences, San Jose, CA, USA).

### Statistical analysis

Quantitative data are expressed as means ± standard deviations. Student’s *t* test or Welch’s t test was used for the statistical analyses, as indicated. Graft survival was compared between the different experimental groups using a Kaplan–Meier analysis. A p value < 0.05 was considered to indicate statistical significance. All statistical calculations were performed using JMP Pro 14.0 (SAS Institute Inc., Cary, NC, USA).

### Ethical approval

Male BALB/c (H-2^d^) and C57BL/6 (B6: H-2^b^) mice were acquired from Japan SLC, Inc. (Shizuoka, Japan). All mice were maintained in a specific pathogen-free environment. The mice were used in experiments when they reached 8–10 weeks of age. The protocols in this study conformed to the guidelines of The Kyoto University Animal Care Committee and were approved by the animal research committee of Kyoto University.

## Supplementary information

Supplementary information

## Data Availability

The datasets analysed during the current study are available from the corresponding author on reasonable request.
