# Supplementary material for: Mitomycin C treatment improves pancreatic islet graft longevity in intraportal islet transplantation by suppressing proinflammatory response
Source: Sci Rep. 2020 Jul 21;10:12086. doi: 10.1038/s41598-020-69009-8 (PMC7374693; doi:10.1038/s41598-020-69009-8)
Supplement: Supplementary file 1 — Supplementary information [file 41598_2020_69009_MOESM1_ESM.pdf]

Supplementary Information

Mitomycin C treatment improves pancreatic islet graft longevity in intraportal islet transplantation by suppressing proinflammatory response

Kei Yamane, Takayuki Anazawa, Seiichiro Tada, Nanae Fujimoto, Kenta Inoguchi, Norio Emoto, Kazuyuki Nagai, Toshihiko Masui, Hideaki Okajima, Kyoichi Takaori, Shoichiro Sumi, Shinji Uemoto

Target	Forward	Reverse
IL-1β	5’-GGATGAGGACATGAGCACCT-3’	5’-AGCTCATATGGGTCCGACAG-3’
MCP-1	5’-TCCCAATGAGTAGGCTGGAG-3’	5’-TCTGGACCCATTCCTTCTTG-3’
IL-6	5’ -GGCCTTCCCTACTTCACAAG-3’	5’ -ATTTCCACGATTTCCCAGAG-3’
TNF-α	5'-AGGGTCTGGGCCATAGAACT-3′	5'-CCACCACGCTCTTCTGTCTAC-3′
INF-γ	5'-ACTGGCAAAAGGATGGTGAC-3′	5'-TGAGCTCATTGAATGCTTGG-3′
IL-2	5'-TGAGCAGGATGGAGAATTACAGG-3'	5'-GTCCAAGTTCATCTTCTAGGCAC-3'
IL-12	5'-TGGTTTGCCATCGTTTTGCTG-3'	5'-ACAGGTGAGGTTCACTGTTTCT-3'
HPRT	5'-CACAGGACTAGAACACCTGC-3'	5'-GCTGGTGAAAAGGACCTCT-3'

Supplementary Table S1. Primers used for quantitative reverse transcription polymerase chain reaction.

Abbreviation: IL-1β, Interleukin-1β; MCP-1, Monocyte Chemotactic Protein-1; IL-6, Interleukin-6; TNF-α, Tumor Necrosis Factor-α; INF-γ, Interferon gamma; IL-2, Interleukin-2; IL-12, Interleukin-12; HPRT, Hypoxanthine phosphoribosyltransferase

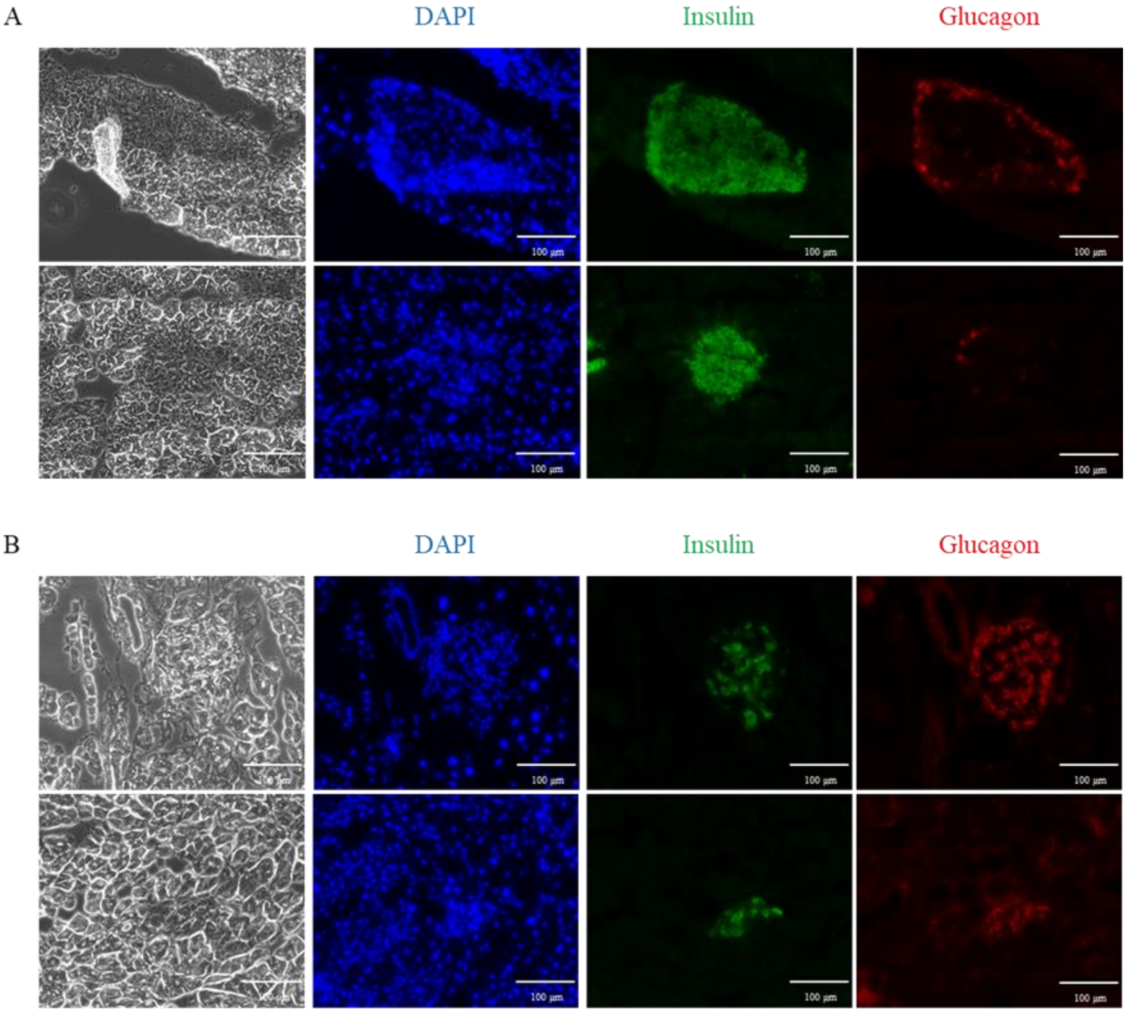

Supplementary Figure S1. Immunofluorescence evaluation of excised pancreatic tissue. Pancreatic tissue from (A) normal healthy mice and (B) transplant recipients were stained with an anti-insulin (1:50 dilution, SC-8033; Santa Cruz Biotechnology, Dallas, TX, USA) and anti-glucagon (1:800 dilution, ab10988; Abcam, Cambridge, UK) at 100 days after the transplantation procedure. The samples were then stained with secondary antibody Alexa Fluor 488 donkey anti-mouse (1:500 dilution, 715-545-150; Jackson ImmunoResearch, West Grove, PA, USA) and Alexa Fluor 594 goat anti-mouse (1:1000 dilution, A-11032, ThermoFisher Scientific, Waltham, MA, USA). Nuclei were counterstained with 4,6-diamidine 2-phenylindoledihydrochloride (DAPI).

A

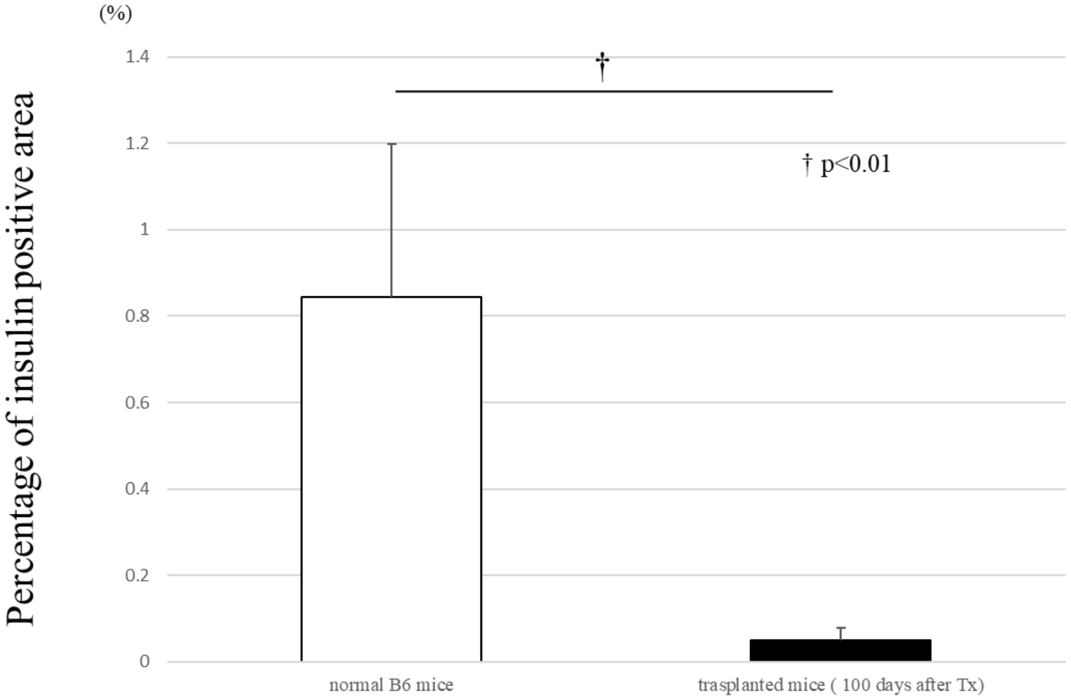

B

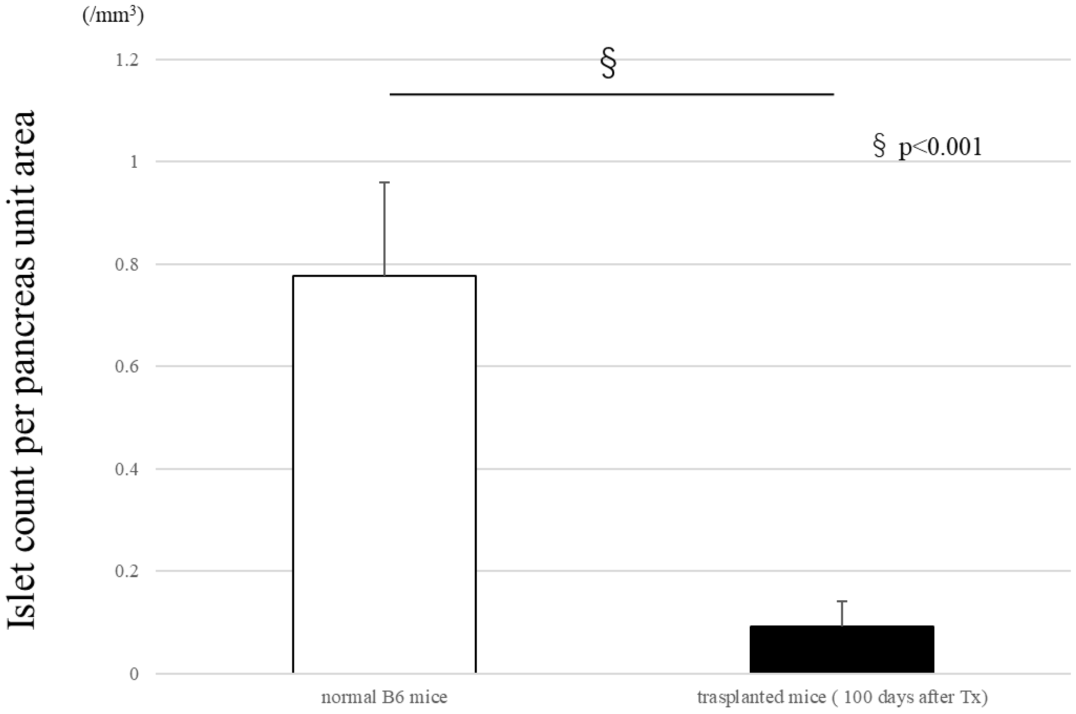

Supplementary Figure S2. (A) The percentage of the total tissue area that stains positive with anti-insulin (n = 4, $^{\dagger}p < 0.01$), and (B) the islet counts per unit area in pancreatic tissue (n = 4, $^{\S}p < 0.001$). The total area and the area staining positive with anti-insulin

were determined using ImageJ software (US National Institutes of Health, Bethesda, MD, USA).
